# MiR-371a-3p Serum Levels Are Increased in Recurrence of Testicular Germ Cell Tumor Patients

**DOI:** 10.3390/ijms19103130

**Published:** 2018-10-12

**Authors:** Angelika Terbuch, Jan B. Adiprasito, Verena Stiegelbauer, Maximilian Seles, Christiane Klec, Georg P. Pichler, Margit Resel, Florian Posch, Anna L. Lembeck, Herbert Stöger, Joanna Szkandera, Karl Pummer, Thomas Bauernhofer, Georg C. Hutterer, Armin Gerger, Michael Stotz, Martin Pichler

**Affiliations:** 1 Division of Clinical Oncology, Department of Internal Medicine, Medical University of Graz, 8036 Graz, Austria; jan.adiprasito@stud.medunigraz.at (J.B.A.); verena.stiegelbauer@gmail.com (V.S.); christiane.klec@medunigraz.at (C.K.); margit.resel@klinikum-graz.at (M.R.); florian.posch@medunigraz.at (F.P.); anna.lembeck@medunigraz.at (A.L.L.); herbert.steoger@medunigraz.at (H.S.); joanna.szkandera@medunigraz.at (J.S.); thomas.bauernhofer@medunigraz.at (T.B.); armin.gerger@medunigraz.at (A.G.); michael.stotz@medunigraz.at (M.S.); 2 Drug Development Unit, Royal Marsden NHS Foundation Trust, Sutton, Surrey SM2 5PT, UK; 3 Research Unit of Non-Coding RNA and Genome Editing in Cancer, Medical University of Graz, 8036 Graz, Austria; 4 Department of Urology, Medical University of Graz, 8036 Graz, Austria; maximilian.seles@medunigraz.at (M.S.); georg.pichler@medunigraz.at (G.P.P.); karl.pummer@medunigraz.at (K.P.); georg.hutterer@medunigraz.at (G.C.H.); 5 Center for Biomarker Research in Medicine (CBmed), 8036 Graz, Austria; 6 Department of Experimental Therapeutics, The University of Texas MD Anderson Cancer Center, Houston, TX 77030, USA

**Keywords:** microRNAs, diagnosis, testicular cancer

## Abstract

Metastatic testicular germ cell tumors (TGCTs) are a potentially curable disease by administration of risk-adapted cytotoxic chemotherapy. Nevertheless, a disease-relapse after curative chemotherapy needs more intensive salvage chemotherapy and significantly worsens the prognosis of TGCT patients. Circulating tumor markers (β-subunit of human chorionic gonadotropin (β-HCG), alpha-Fetoprotein (AFP), and Lactate Dehydrogenase (LDH)) are frequently used for monitoring disease recurrence in TGCT patients, though they lack diagnostic sensitivity and specificity. Increasing evidence suggests that serum levels of stem cell-associated microRNAs (miR-371a-3p and miR-302/367 cluster) are outperforming the traditional tumor markers in terms of sensitivity to detect newly diagnosed TGCT patients. The aim of this study was to investigate whether these miRNAs are also informative in detection of disease recurrence in TGCT patients after curative first line therapy. For this purpose, we measured the serum levels of miR-371a-3p and miR-367 in 52 samples of ten TGCT patients at different time points during disease relapse and during salvage chemotherapy. In our study, miR-371a-3p levels in serum samples with proven disease recurrence were 13.65 fold higher than levels from the same patients without evidence of disease (*p* = 0.014). In contrast, miR-367 levels were not different in these patient groups (*p* = 0.985). In conclusion, miR-371a-3p is a sensitive and potentially novel biomarker for detecting disease relapse in TGCT patients. This promising biomarker should be investigated in further large prospective trials.

## 1. Introduction

Testicular germ cell tumors (TGCTs) represent the most common malignancy among men aged between 20 and 40 years [1]. In oncology practice, TGCTs are sub-classified according to their underlying histology into seminomatous (SGCT) and non-seminomatous germ cell tumors (NSGCT), which are distinct in terms of biology, prognosis, and treatment strategy. In metastatic TGCTs, prognosis and therapy depend on the size and location of metastases, as well as levels of circulating tumor markers. According to the International Germ Cell Cancer Consensus Group (IGCCCG) recommendations, TGCT patients are stratified into different prognosis groups [2]. For follow-up monitoring to detect disease relapse, blood-based tumor markers alpha-Fetoprotein (AFP), the β-subunit of human chorionic gonadotropin (β-HCG), and Lactate Dehydrogenase (LDH) are commonly measured [3]. However, these tumor markers possess a limited sensitivity with a no more than 60 percent positive detection rate in all TGCT patients [4]. Even worse, in pure seminoma patients, β-HCG is only useful in less than 20% of patients [5]. Taken together, especially in seminoma patients, the discovery and implementation of novel serum-based tumor markers might be helpful for early diagnosis and monitoring of disease relapse. Beyond the protein-based tumor markers, a huge group of RNA molecules, so called non-coding RNAs, show great potential as biomarkers [6]. In general, non-coding RNAs are divided by their length in long (>200 nucleotides) and small (<200 nucleotides) non-coding RNAs [7].

MicroRNAs (miRNAs) are small RNA molecules, approximately 22 nucleotides in length, with a non-coding nature [8]. They are single-stranded RNAs that are known to influence post-transcriptional gene expression through binding and interaction with their target messenger RNAs (mRNAs), through complementary sequences in the 3′-untranslated region. Thereby, microRNAs suppress the protein translation or initiate degradation of the mRNA by the RNA interference effector complex [9]. MiRNAs have been demonstrated to carry great potential as stable and measurable biomarkers in tissue and body fluids [8,10,11,12]. Over recent years, many studies have found altered miRNA expression in different types of malignancies, suggesting their impact in carcinogenesis as tumor suppressor miRNAs as well as oncogenic miRNAs [13,14,15,16,17]. For example, miRNAs are differentially expressed in various soft tissue sarcoma subtypes, and therefore show potential as diagnostic tools in challenging cases [18]. For testicular cancer, a growing body of evidence proposes miR-371a-3p and miR-367 to be far more sensitive and specific diagnostic markers for detection of primary TGCT compared to the traditional serum markers AFP, β-HCG, and LDH [19,20]. For instance, Gillis et al. have shown that in 80 TGCT patients the serum levels of miR-371a-3p and miR-367 were significantly higher than in healthy controls [21]. In patients with localized disease, the serum miR-371a-3p and miR-367 levels were significantly lower after surgical removal of the tumor-bearing organ compared with serum samples before the treatment [21]. A comparison with traditional serum tumor markers showed a sensitivity of 98% for miR-371a-3p and miR-367 levels compared to a sensitivity of 50% for AFP and β-HCG [21]. Furthermore, it was demonstrated that they are specific to TGCTs, as they are absent in other malignancies [22].

In a prospectively conducted biomarker study, Dieckmann et al. clearly demonstrated that miR-371a-3p has the best discriminative power with 88.7% sensitivity and 93.4% specificity, outperforming AFP, β-HCG, and LDH which showed a combined 50% sensitivity [19]. Despite this encouraging data in the setting of detection of primary TGCTs and disease monitoring, very little information about the value of these miRNAs in TGCT disease-recurrence is published. Thus, the aim of this study was to test the hypothesis that embryonic stem cell associated miRNAs are appropriate serum biomarkers for indicating recurrence of TGCTs.

## 2. Results

The median time from serum sample collection to the miRNA analysis was 12 years (interquartile range: 4.8 years to 14 years, range: minimum of 7 months to maximum of 16.9 years). No significant correlation between the duration of storage (age of serum sample) and levels of miR-371 and miR-367 after normalization, or cycle threshold (CT)-values of reference miRNAs (*p* > 0.05), was observed, indirectly indicating a high chemical stability for these molecules. In the next step we were interested in whether miRNA levels were differentially expressed according to the pathological confirmed histologic subtypes. One patient with pure teratoma had no measurable miRNA levels. Comparing the miRNAs and traditional tumor markers in serum samples from patients with seminomatous and non-seminomatous TGCTs indicated that AFP (*p* < 0.001) and β-HCG (*p* = 0.004) were aberrant (increased) in non-seminoma patients. There was no significant correlation between miR-371a-3p and miR-367-3p, AFP, β-HCG, and LDH serum levels.

**Figure 1:**
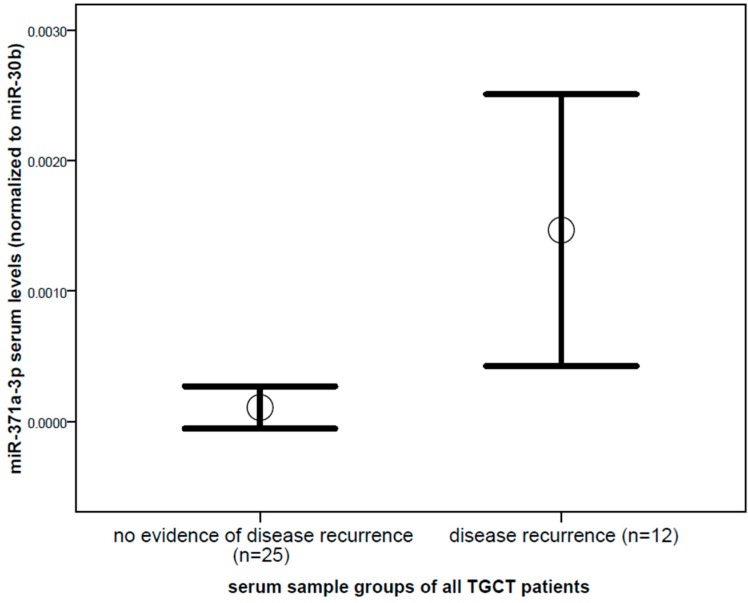
Comparison of miR-371a-3p levels between serum samples of all patients with no evidence of disease (*n* = 25) and serum samples of patients with proven and yet untreated disease recurrence (*n* = 12) of testicular germ cell cancer of all histologies. Mean values (circles) and 1.5 times standard error of the mean (bars) are depicted (13.65 fold difference, *p* = 0.014). MicroRNA (miRNA) level is shown as a normalized log2 transformed value normalized to miR-30b-5p.

**Figure 2:**
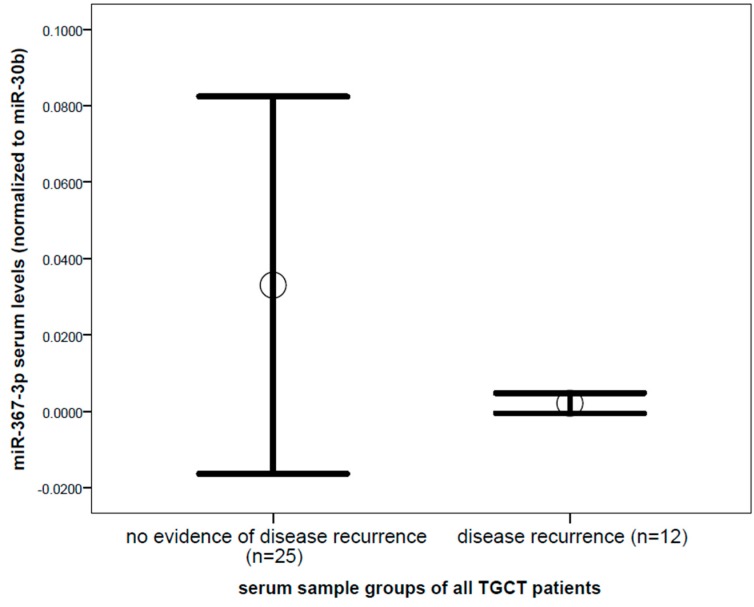
Comparison of miR-367 levels between serum samples of all patients with no evidence of disease (*n* = 25), and serum samples of patients with proven and yet untreated disease recurrence (*n* = 12) of testicular germ cell cancer of all histologies, did not show a significant difference. Mean values (circles) and 1.5 times standard error of the mean (bars) are depicted (*p* = 0.985). MiRNA level is shown as a normalized log2 transformed value normalized to miR-30b-5p.

Comparing miR-371a-3p levels between serum samples of untreated patients with proven disease recurrence, and serum samples from the same patients without evidence of disease, showed a significantly increased mean value (13.65 fold difference, *p* = 0.014, Mann–Whitney *U* test) in the group of proven disease recurrence (Figure 1). In contrast, miR-367-3p did not show a significant difference between serum samples of patients with no evidence of disease and proven recurrence (*p* = 0.985, Figure 2). With regard to the traditional tumor markers, only β-HCG levels were significantly different in serum samples of proven disease recurrence (2.4 fold increase, *p* = 0.025, Mann–Whitney *U* test) compared to serum samples of patients with no evidence of disease, whereas no significant difference was found for AFP (*p* = 0.795) and LDH levels (*p* = 0.827). When comparing the serum levels of the miRNAs in samples of proven disease recurrence (*n* = 12) before treatment start and samples retrieved during the salvage chemotherapy (*n* = 15) from the same patients, a trend of decline in levels of miR-371-3 was observed, though due to the small sample size, this difference did not reach statistical significance.

**Figure 3:**
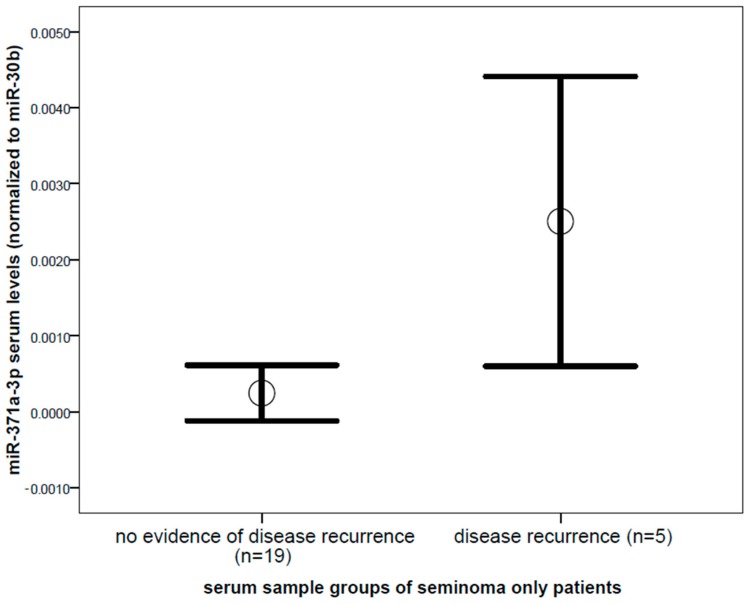
Comparison of miR-371a-3p levels between serum samples of seminoma patients with no evidence of disease (*n* = 19) and serum samples of patients with proven and yet untreated disease recurrence (*n* = 5) of testicular germ cell cancer. Mean values (circles) and 1.5 times standard error of the mean (bars) are depicted (*p* = 0.031). MiRNA level is shown as a normalized log2 transformed value normalized to miR-30b-5p.

Focusing on serum samples from seminoma patients only (*n* = 24), we found significantly increased levels of miR-371a-3p (10.2 fold, *p* = 0.031, Mann–Whitney *U* test, Figure 3) in serum from patients with proven disease recurrence compared to no-evidence-of-disease samples. Other markers, including miR-367-3p, AFP, β-HCG, and LDH, were not significantly different in serum samples of seminoma patients in disease recurrence compared to no-evidence-of-disease samples (*p* > 0.05).

**Figure 4:**
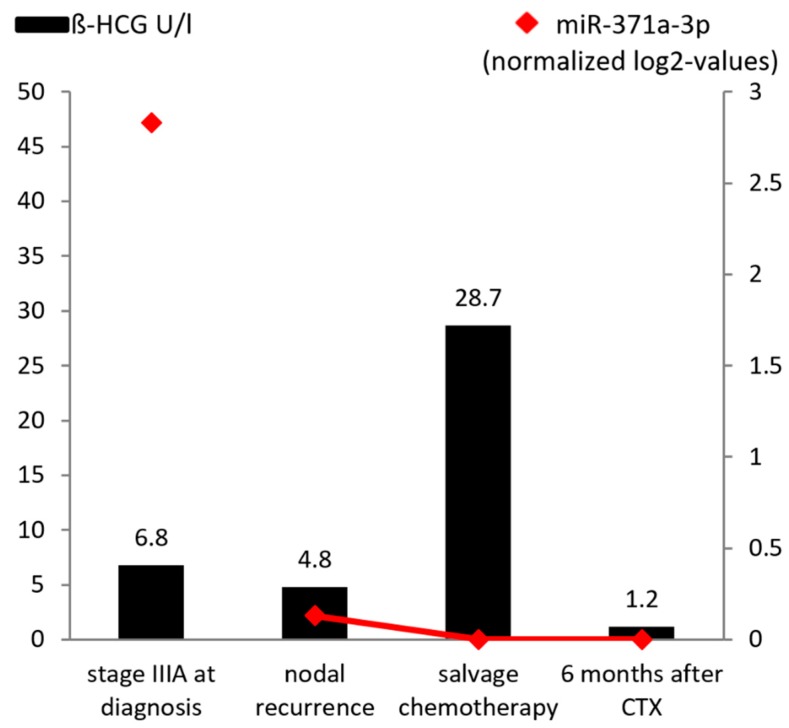
Representative example of an individual course of miR-371a-3p serum levels in a seminoma patient at initial diagnosis (stage IIIA), at the time of relapse (lymph node metastases), during salvage chemotherapy, and at 6-month-follow-up with no evidence of disease. MiRNA level is shown as a normalized log2 transformed value normalized to miR-30b-5p on a 10^−3^ scale.

Figure 4 shows miR-371a-3p levels during the disease course of a seminoma patient including serum levels of β-HCG. It reflects the very high level of miR-371a-3p corresponding to a high tumor burden at initial diagnosis with stage IIIA disease. At the time of retroperitoneal lymph node metastases relapse, the miR-371a-3p level was much lower than at the time of primary diagnosis. As we did not collect a serum sample after first line chemotherapy and nodal recurrence, we did not connect the first measurement to the red line in the figure. It might be that the miR level became undetectable after first line chemotherapy and increased at time of nodal recurrence, but there is also the possibility that the miR level never became completely negative, indicating residual disease. During curative chemotherapy for nodal recurrence, the miR-371a-3p level decreased continuously and was not detectable after completion of treatment. In comparison, β-HCG levels increased during salvage treatment, which we retrospectively identified as a ‘false-positive’ tumor marker rise, probably due to destruction of cancer cells during chemotherapy. For the non-seminoma samples, we could not detect miR-371 levels in samples without evidence of disease recurrence. We therefore did not want to perform a separate analysis in the non-seminoma samples.

## 3. Discussion

TGCTs are unique in terms of high cure rates, even in patients with metastatic disease after treatment with orchiectomy and curative chemotherapy. However, depending on tumor stage, a low percentage of patients experience disease recurrence with a potential risk of chemo-resistance, chemotherapy-associated toxicity, and cancer related death [23]. Sensitive and specific biomarkers could simplify follow-up and reduce the number of radiological examinations. The detection of miR-371a-3p and miR-367-3p in serum has been reported to be sensitive and specific for diagnosis of primary TGCTs, and a correlation between serum level and clinical stage has been demonstrated in multiple studies [4,19,24,25,26,27]. For instance, Belge and colleagues compared the serum level of miR-371-3p before and after orchiectomy in TGCT patients, and found that postoperative levels were significantly lower in all cases [28]. Dieckmann et al. investigated the miRNA serum levels during chemotherapy for TGCT patients with advanced disease, and showed that the serum levels dropped to normal after completion of chemotherapy [19,29]. In their study, they also reported about nine patients with disease-recurrence and suggested the potential value of miR-371-3p in the setting of disease recurrence [19]. In addition, another small study suggested that these miRNAs are more sensitive for detecting relapse and residual disease in TGCT patients than the traditional tumor markers, by showing the individual course of six TGCT patients during follow-up [24]. However, only one patient with seminomatous histology was included in this case collection. Particularly in seminoma patients, new biomarkers would be helpful as the traditional tumor markers are only useful in about 20% of patients.

In our study, we retrospectively investigated serum samples of ten TGCT patients, including four with pure seminomatous histology. MiR-371a-3p performed significantly better than miR-367-3p to diagnose disease recurrence in TGCT patients, and based on our data and that of others, should be the miRNA of choice for further testing in prospective biomarker trials.

Furthermore, we were able to show that the levels of miR-371a-3p were 10 fold higher in seminoma patients with proven recurrence compared to samples at the time of no evidence of disease, suggesting miR-371a-3p as a potential serum biomarker, especially for seminoma patients. Furthermore, our results showed that the levels of miR-371a-3p declined during treatment with chemotherapy. Unfortunately, blood samples were not stored throughout the whole course of disease for every patient. However, the decline in miRNA levels was numerically greater in patients who responded to chemotherapy. Due to the small sample size, the difference did not reach statistical significance and should therefore be externally validated in larger cohorts.

In line with other studies, we could not detect miRNA levels in teratoma serum samples. MiR-371a-3p and miR-367 are epigenetically silenced in adult somatic cells, and also in teratoma, which represent the group of GCTs with the highest degree of maturation [30]. However, Vilela-Salgueiro et al. recently demonstrated that miR371a-3p levels were higher in teratoma tissue samples when compared to healthy testis tissue [26]. Interestingly, in a retrospective cohort study by Leão and colleagues, miR371a-3p levels in serum were shown to accurately predict viable disease after chemotherapy in patients with NSGCT. This finding is intriguing, as miR-371a-3p carries the potential to spare patients from unnecessary post-chemotherapy retroperitoneal lymph node resection. In line with our study where we could not detect miR-371a-3p in serum from a teratoma patient, miR371a-3p was not able to distinguish between the presence of fibrosis or teratoma [31]. This lack of miRNAs might still be helpful for the diagnosis of a growing teratoma phenomenon. As we have shown that miRNAs have the potential to indicate recurrent disease, a growing teratoma syndrome is likely in growing lesions without increased miRNA serum levels. As chemotherapy is not effective in patients with growing teratoma, these patients need to undergo surgery. If miRNA levels are still detectable after chemotherapy, salvage-chemotherapy under close monitoring of miRNA levels might be an alternative approach before undergoing surgery. However, this needs to be investigated in prospective trials, but might be one role for miRNAs in the future.

Our study is not without limitations due to the retrospective design, and therefore a possible selection bias cannot be ruled out. A further limitation of our analysis is the lack of a formal control group of healthy men. The cohort, though one of the largest ones reported yet, is rather small and underpowered to detect significant differences in specific sub-groups like histology. The samples were collected over a long period of time, the pathology reports were not centrally assessed by the same pathologists, and the salvage treatment included different types of chemotherapeutic strategies. Protocols may differ between laboratories, and especially as we used samples with many years of storage and no standardized pre-analytical procedures, we cannot rule out miRNA degradation in our samples, which might lead to the lower amounts of detection. A gold standard of how to use controls, protocols, and PCR reagents with this biomarker still has to be defined, though some studies were using an external xenomiR to calibrate and adjust for losses of RNA and incongruences in cDNA synthesis.

In conclusion, we have shown that miRNAs might be novel biomarkers for detection of disease recurrence in TGCT patients. In patients with seminoma, where the classical serum markers are not helpful in the majority of patients, this new biomarker is promising to outperform current state-of-the-art serum diagnostics. Based on our data and several lines of evidence in the literature, miR-371a-3p should be the miRNA of choice for further testing. Our findings warrant further investigation in prospective trials, but miRNAs seem to have the potential to improve diagnosis and follow-up of TGCT patients.

## 4. Materials and Methods

In this study we analyzed 52 serum samples from ten patients with recurrent TGCTs after failure of curative first line therapy. The patients’ clinico-pathological data was retrieved from a dataset including 950 consecutive patients with histologically confirmed TGCTs, presenting to the Division of Oncology at the Medical University of Graz between January 1994 and December 2013 (Table 1) [32,33]. Patients were initially staged using computed tomographic (CT) scans of the abdomen, CT scans or X-rays of the chest, and postoperative tumor markers α-fetoprotein (AFP), human chorionic gonadotropin (HCG), and lactate dehydrogenase (LDH). Patients’ records were retrospectively reviewed, and all patients were classified according to the 7th TNM staging system and the International Germ Cell Cancer Collaborative Group (IGCCCG) classification [2,34]. Follow-up data was retrieved until January 2015. Follow up investigations at our center were performed according to a local protocol, and were adapted in 2007 and 2012 according to recent publications [35]. Of this overall cohort, ten patients with recurrent disease and available stored samples were identified. Disease recurrence was defined as either radiological relapse or marker increase (one patient), which can be seen in Table 1. Six patients had NSGCT and four patients had SGCT (Table 1). Of the patients with NSGCT, case 1 represented mixed histology (equally distributed between yolk, teratoma, and seminoma), case 2 had mixed histology as well (equally distributed between embryonal, yolk, teratoma, and seminoma), case 4 had predominantly histology of choriocarcinoma, case 8 represented a yolk sac tumor, case 9 had mixed histology with 60% embryonal carcinoma, and case 10 had pure embryonal carcinoma. We categorized our serum samples into three different groups: (1) Twelve serum samples of patients with newly diagnosed and untreated recurrent metastatic TGCTs, (2) fifteen serum samples during chemotherapeutic treatment of recurrent metastatic TGCTs, and (3) twenty-five serum samples from follow-up with no evidence of biochemical or radiographic disease of these patients. Serum tumor markers were measured at the same time points as miRNA levels. This study was approved by the local ethical committee of the Medical University of Graz (28-449 ex 15/16).

### MiRNA Isolation, cDNA Synthesis, and Quantitative RT PCR (qRT-PCR)

For miRNA isolation from serum samples, a miRNeasy Kit (Qiagen, Hilden, Germany) was used to extract total RNA from 200 µL of serum. A three-step procedure was performed to measure miRNA expression in human serum samples. For cDNA synthesis, 50 ng of total RNA was subjected to Reverse Transcription (RT) using the TaqMan microRNA Reverse Transcription Kit (Thermo Fisher, Waltham, MA, USA) and a pool containing four specific 5× RT primers (TaqMan miRNA Assay specific for miR-371a-3p, miR-367-3p, miR-93-5p, and miR30b-5p, Thermo Fisher, Waltham, MA, USA) following the manufacturer’s protocol, allowing simultaneous reverse transcription of four miRNAs of interest [4,19,24]. RT was performed on a MyCycler thermocycler (Biorad, Hercules, CA, USA) according to the manufacturer’s recommendations. Afterwards, a pre-amplification step was performed using a pre-amp Primer pool of four specific 20× TaqMan miRNA Assays (TaqMan miRNA Assay specific for miR-371a-3p, miR-367-3p, miR-93-5p, and miR30b-5p, Thermo Fisher) and the TaqMan PreAmp Mastermix (Thermo Fisher) following the manufacturer’s instructions. In detail, for each reaction 3.12 µL of RT product was mixed with 6.25 µL of TaqMan PreAmp Mastermix and 3.12 µL of 100-fold diluted pre-amp Primer pool. Pre-amp reactions were performed on a MyCycler thermocycler (Biorad). We used a pre-amplification protocol of 10 cycles followed by a quantitative real-time PCR approach of 35 cycles. For quantitative RT-PCR, pre-amplified PCR products were five-fold diluted using RNAse free water, and relative quantification of miRNAs was performed using TaqMan Universal Mastermix II No UNG (Thermo Fisher) and specific 20× TaqMan miRNA Assays on a Light Cycler 480 real-time PCR device (Roche Applied Science, Mannheim, Germany). Expression values were calculated using normalization to miR-93-5p and miR30b-5p (after the formula 2^–(target miRNA–miRNA reference)), and further used for statistical analysis.

Based on previous reports we selected the two most promising miRNA candidates (miR-371a-3p and miR-367-3p). For normalization of the levels of these serum microRNAs, we tested two previously described reference miRNAs (miR-30b-5p and miR-93), though one of them, miR-93, was shown to be not optimal in a recently published study [24,30]. We observed a significant (R = 0.438, *p* = 0.001) Spearman-correlation of these reference miRNAs in our serum samples. In order to avoid misinterpretation of results depending on the reference miRNA used, we decided to use both strategies (the geometric mean for miR-30b-5p and miR-93, as well as miR-30b-5p only) for normalization. For reasons of clarity, we report here only the results generated by using miR-30b-5p only as the reference, as previously reported (Supplementary Materials) [24,30]. However, in case of varying results with regard to significance (*p* < 0.05), we explicitly mention such findings. Data is shown as relative comparisons in error bars including a 1.5 fold standard error of the mean interval. The non-parametric Mann–Whitney *U* test was used to test for statistical significance by the SPSS software version 18 package (IBM Corp., Armonk, NY, USA).

## Figures and Tables

**Table 1:** Clinicopathological characteristics. MFS: metastasis-free-survival (months). CSO: cancer specific outcome (D: dead, CR: complete remission). * Age in years; ** Serum tumor markers at time of recurrence, during curative treatment, and during follow-up; brackets indicate reference ranges. NA: not applicable due to missing values.

Case	Age *	Histol.	Primary Stage	MFS	Site of Relapse	CSO	β-HCG (0–5 U/L) **			AFP (0–15 ng/mL) **			LDH (120–240 U/L) **		
							REC	CTX	FU	REC	CTX	FU	REC	CTX	FU
1	38	NS	IIIC	2	LN retrop	D	<1.2	<1.2	<1.2	210	229	14	156	262	175
2	45	NS	IIIC	72	LN med	D	<1.2	NA	NA	9.5	NA	NA	192	NA	NA
3	70	S	IIB	2	LN retrop	D	<1.2	<1.2	<1.2	3.4	3.3	3.6	163	162	163
4	27	NS	IIIC	22	brain	D	NA	NA	5	NA	NA	3.7	NA	NA	135
5	39	S	IIB	3	LN retrop	CR	4.8	28.7	<1.2	1.6	2.0	2.2	235	243	370
6	45	S	IIA	11	LN med	CR	1.7	<1.2	<1.2	5.8	4.7	4.3	295	196	188
7	29	S	IIB	5	lung	D	2.4	NA	NA	2.0	NA	NA	199	NA	NA
8	42	NS	IIIC	2	liver	D	<1.2	NA	NA	1652.3	NA	NA	191	NA	NA
9	30	NS	IIB	18	LN retrop	CR	NA	NA	<1.2	NA	NA	4.2	NA	NA	189
10	48	NS	IIA	1	markers	CR	<1.2	<1.2	<1.2	244.6	151.1	110.7	174	174	153

## Supplementary Materials

Supplementary materials can be found at http://www.mdpi.com/1422-0067/19/10/3130/s1.
