# Supplementary material for: MiR-371a-3p Serum Levels Are Increased in Recurrence of Testicular Germ Cell Tumor Patients
Source: Int J Mol Sci. 2018 Oct 12;19(10):3130. doi: 10.3390/ijms19103130 (PMC6213366; doi:10.3390/ijms19103130)
Supplement: Supplementary file 1 [file ijms-19-03130-s001.pdf]

Sample	Date of Sample Collection	AFP	betaHCG	LDH
1	11.02.2002	1288.7	0	240
2	11.09.2001	1897.8	0	196
3	03.10.2001	1614	0	167
4	06.01.2002	305.7	0	157
5	16.03.2001	887	0	154
6	08.05.2001	210	0.9	156
8	27.07.2000	14.4	0	175
9	29.11.2000	-9	-9	135
10	15.12.2000	229	0	262
11	08.09.2000	8.8	1.2	176
13	16.08.2005	9.1	1.2	200
14	12.08.2009	7.4	1.2	215
15	03.03.2010	7.7	1.2	189
16	01.09.2010	6.6	1.2	174
17	17.12.2010	9.5	1.2	192
18	18.02.2011	7.4	1.2	190
19	25.04.2012	3.4	1.2	163
20	14.01.2013	3.3	1.2	162
21	16.04.2013	3.6	1.2	163
22	30.07.2013	3.1	1.2	165
23	04.11.2013	2.7	1.2	187
24	31.01.2014	3.6	2.8	152
25	01.08.2012	3.7	5	135
26	31.08.2012	4.8	4.8	185
27	17.06.2013	1.6	6.8	235
28	04.03.2004	2	4.8	243
29	21.07.2004	2.9	28.7	216
30	11.08.2004	3.6	1.2	360
31	22.09.2004	2.5	1.2	151
32	14.02.2005	2.2	1.2	370
33	02.05.2005	4.6	1.2	140
38	14.07.2003	5.1	2.4	148
39	20.01.2004	5.8	1.7	295
40	27.04.2004	4.7	1.2	196
41	11.06.2004	4.3	1.2	188
42	01.09.2004	5.1	1.2	147
43	04.11.2004	5.1	1.2	140
44	08.06.2005	-9	-9	-9
45	28.07.2005	5	1.2	210
46	04.03.2011	151.1	1.2	174
47	12.08.2016	110.7	1.2	153
48	11.11.2016	2	2.4	199
51	18.03.2003	-9	-9	-9
52	06.05.2003	-9	-9	-9
53	09.07.2003	1652.3	0	191
55	30.06.2000	4.2	1.2	189
56	11.11.2010	4.6	1.2	200

57	06.05.2011	5.2	1.2	204
58	03.11.2011	5.3	1.2	251
59	19.12.2014	235.6	-9	195
60	03.06.2016	244.6	1.2	174
61	23.06.2016			

Disease status (1 = no disease, 2 = disease under treatment, 3 = untreated recurred disease)
2
2
2
2
2
2
3
1
2
2
1
1
1
1
1
3
3
3
1
1
1
3
3
3
3
3
2
2
2
1
1
1
3
3
2
1
1
1
1
1
1
1
2
2
2
3
1
1

1
1
1
1

miR371-miR30b)	miR367-miR30b)	Histology
0	0.0221738	NSGCT
0	0.00030583	NSGCT
0	0.0003513	NSGCT
0	0	NSGCT
0	0.00013925	NSGCT
0	0	NSGCT
0	0	NSGCT
0	0	NSGCT
0	0	NSGCT
0.00088625	0	NSGCT
0	0	NSGCT
0	0	NSGCT
0	0.00004286	NSGCT
0	0.00018761	NSGCT
0	0.00002877	NSGCT
0	0	NSGCT
0	0	NSGCT
0	0	SGCT
0	0.00012335	SGCT
0	0	SGCT
0	0	SGCT
0.00587987	0	SGCT
0	0.00004546	NSGCT
0	0.00007998	NSGCT
0	0.00007567	NSGCT
0.00144973	0	SGCT
0.00005636	0	SGCT
0	0.00010159	SGCT
0	0.00015886	SGCT
0	0	SGCT
0	0	SGCT
0	0	SGCT
0.00519018	1813065.984	SGCT
0	0	SGCT
0	345901.0818	SGCT
0	0	SGCT
0.00268661	0	SGCT
0	1819360.493	SGCT
0	661325.0389	SGCT
0	565826.4137	SGCT
0	0	NSGCT
0	0	NSGCT
0.00754638	0.00319493	SGCT
0	0.82359102	SGCT
0	0.00341239	SGCT
0.00508337	0.0209777	NSGCT
0	0.00007752	NSGCT

0	0	NSGCT
0	0.00106495	NSGCT
0	0.00280069	NSGCT
0	0	NSGCT
0	0	NSGCT
